# Persistence of Symptoms Following Infection With COVID-19 Among Patients With Type 1 and Type 2 Diabetes Mellitus in Saudi Arabia

**DOI:** 10.7759/cureus.43556

**Published:** 2023-08-16

**Authors:** Maram H Abduljabbar, Ghadeer A Alhawsawi, Sarah S Aldharman, Khawlah I Alshahrani, Razan A Alshehri, Abdulmajeed A Alshehri, Abdulrahman T Abukhudair, Maram A Alghamdi

**Affiliations:** 1 Department of Pharmacology and Toxicology, College of Pharmacy, Taif University, Taif, SAU; 2 College of Pharmacy, Taif University, Taif, SAU; 3 College of Medicine, King Saud Bin Abdulaziz University for Health Sciences, Riyadh, SAU; 4 College of Medicine, King Abdulaziz University, Rabigh, SAU

**Keywords:** infection, saudi arabia, type 2 diabetes mellitus, type 1 diabetes mellitus, covid-19

## Abstract

Background: In Saudi Arabia, information on the persistence of COVID-19-related complications in diabetic patients, their associations with the type of diabetes mellitus (DM), and the role of uncontrolled DM in the complications remains elusive. This study aims to fill this gap. This study aimed to examine the persistence of COVID-19 complications in diabetic patients.

Method: A simple randomized, cross-sectional, questionnaire-based study among patients with type 1 and 2 DM following infection with COVID-19 in Saudi Arabia.

Result: In the present study, a total of 674 participants were included. Among the COVID-19 symptoms, fatigue (65.6%) was reported the most frequently, followed by headache (62.3%) and cough (59.8%). About 44% of patients reported high blood sugar levels, including 25.5% with elevated fasting (>126 mg/dL) and 18.5% with elevated postprandial (>200 mg/dL) glucose levels. We also report that age > 55 years [OR= 1.66 (1.01-2.56), p=0.045], anti-diabetic medications [OR= 2.10 (1.82-3.91), p=0.022], multiple comorbidities [OR= 3.21 (1.98-4.85), p=0.005], chest pain [OR= 2.54 (0.96-3.81), p=0.003], and joint pain [OR= 1.64 (0.73-2.94), p=0.025] were independently associated with COVID-19-related complications in diabetic patients.

Conclusion: The most common persistent symptoms in diabetic patients with COVID-19 infection are fatigue, headaches, and cough. Advanced age and use of antidiabetic medications were independently associated with COVID-19-related complications in diabetic patients.

## Introduction

Newly developing viral illnesses have become a major public health concern worldwide in recent years. Several viral infections have been reported in the past two decades, including SARS-CoV, H1N1 influenza, MERS-CoV, Ebola virus disease (EVD), and Zika virus [1]. The most recent viral illness produced by a new coronavirus has affected public health everywhere. The Severe Acute Respiratory Syndrome Coronavirus-2 (SARS-CoV-2) was named by the International Committee on Taxonomy of Viruses because of its resemblance to the preceding SARS-CoV [2]. Later, the World Health Organization (WHO) labeled this illness a coronavirus disease in 2019 (COVID-19) [3]. The SARS-CoV-2 virus outbreak quickly spread throughout numerous countries on March 11, 2020, prompting the WHO to declare COVID-19 a worldwide pandemic [4]. While all coronaviruses primarily target the respiratory system, the SARS-CoV-2 virus also attacks the heart, gastrointestinal system, liver, kidneys, and central nervous system, resulting in multi-organ failure [5]. Patients with COVID-19 may experience a variety of symptoms, but the most common clinical signs are fever, tiredness, coughing up sputum, anorexia, and shortness of breath [6,7]. Diabetes mellitus (DM) is a serious public health issue marked by metabolic derangements and dysregulated glucose homeostasis due to insufficient insulin secretion and/or insulin resistance. Diabetes and its complications are among the leading causes of death worldwide [6,8]. The prevalence of DM has increased over the last decade due to increased life expectancy, unhealthy food habits, sedentary lifestyles, and increased obesity due to urbanization [8]. DM is a long-term metabolic condition marked by persistent hyperglycemia [9]. The burden of diabetes is high and increasing in all countries due to the increasing global prevalence of obesity and unhealthy lifestyles. Diabetes is generally classified into type 1 diabetes (T1D) and type 2 diabetes (T2D), with the latter type accounting for most diabetes cases (>85%) [6,8,9,10]. Common complications of DM include microvascular endpoints such as retinopathy, nephropathy, and neuropathy, as well as macrovascular endpoints such as ischemic heart disease, stroke, and peripheral vascular disease [10]. Diabetes is a serious public health problem because of its morbidity, mortality, reduced life expectancy, and financial and other costs [11]. Type 1, type 2, Maturity Onset Diabetes of the Young (MODY), neonatal diabetes, steroid-induced diabetes, and gestational diabetes are just a few of the subtypes [11]. Type 1 and 2 diabetes mellitus are the two most common subtypes, each having its own pathogenesis, presentation, and care, yet all can result in hyperglycemia [12]. In the United States, diabetes is the leading cause of end-stage renal disease, nontraumatic amputation, and new-onset blindness. Cardiovascular complications account for up to 80% of all mortalities among diabetic patients [12]. Among the tactics employed to combat diabetic phenotypes are hygiene measures (diet and exercise) and vigorous treatment of hypertension, dyslipidemia, and hyperglycemia [13].

Patients with diabetes mellitus are more likely to contract the coronavirus disease 2019 (COVID-19), which causes severe acute respiratory syndrome (SARS-CoV-2) [14]. Infected individuals who have COVID-19 may have an increased risk of hyperglycemia [14,15]. Hyperglycemia may inhibit immune and inflammatory responses when combined with other risk factors, putting people at risk for serious COVID-19 and perhaps fatal results [15]. DM is associated with an increased risk of complications, extended hospital admissions, and mortality in patients with COVID-19 infection [14,16]. Patients with hyperglycemia on COVID-19 display severe clinical problems, more ICU hospitalizations, need mechanical ventilation, and exhibit a substantial rise in inflammatory markers [14-16]. The risk of death and COVID-19 severity are two to four times higher in diabetic or hyperglycemic patients than in nondiabetic patients [17]. Several factors influence the severity and mortality of infection in people with underlying health issues such as DM [17]. Patients in the high-risk group contracting the infection, such as diabetics, have a significant risk of death or developing severe complications [18]. One of the symptoms or problems that might appear in DM patients with COVID-19 infection is called post-COVID-19 syndrome, which is defined as symptoms or problems that persist for 12 weeks or longer after taking COVID-19 [19]. The most prevalent and long-lasting symptom in diabetic patients is exhaustion, which shares similarities with chronic fatigue syndrome [19]. Furthermore, during the COVID-19 pandemic, individuals previously diagnosed with diabetes demonstrated poor diabetes control, and there was a high rate of new-onset diabetes, which could be attributed to the increased use of steroids for COVID-19 treatment, all of which led to higher morbidity and mortality [19,20]. Patients with diabetes are at a higher risk of developing viruses, especially Type 2 diabetics, due to immune deficiencies. Consequently, these patients develop serious COVID-19-related risks and symptoms. According to the CDC, people with T1D and T2D are more likely to get a COVID-19 infection [20,16].

Patients with diabetes who have a COVID-19 infection are at significant risk of having a stroke or having their blood supply to their legs reduced. The patient may have specific symptoms such as headache, fever, joint pain, and muscle aching [21]. After being infected with COVID-19, a patient is at risk of diabetic ketoacidosis (DKA), especially in patients with T1D [22]. DKA is most common in overweight patients with DM [22].

Despite these findings, the types and duration of the persistence of COVID-19-related complications in DM patients are poorly known. It is also not well characterized if the presence of uncontrolled vs. controlled diabetes can significantly affect these complications. Additionally, the associations of T1D and T2D with these complications partly remain elusive. We aimed to answer these questions by investigating the associations between COVID-19-related complications and patients with DM. We hypothesize that the presence of uncontrolled DM and advancing age are associated with higher severity and prolonged persistence of COVID-19-related complications in DM patients. This study aimed to examine the persistence of COVID-19 complications and their associated factors among patients with Type 1 and Type 2 Diabetes Mellitus (DM) in Saudi Arabia. 

## Materials and methods

Study design

A randomized, cross-sectional study was performed through an online questionnaire among patients with type 1 and type 2 diabetes mellitus following infection with COVID-19 people in Saudi Arabia. A self-administered online survey was applied to patients with type 1 and type 2 diabetes mellitus (created using Google Forms). The purpose of the study was to encourage participants by directing them to a description page before obtaining informed consent. The voluntary participation and anonymity statement were also included. The research ethics committee at Taif University, Saudi Arabia, approved this study [approval number: (HAO-02-T-105)].

Collection tool

After an extensive literature review, the author designed a questionnaire applicable to the study objectives. The questionnaire was about the persistent complications of COVID-19 in diabetic patients. The questionnaire consists of 17 questions, categorized into three sections. The first section contained demographic information, the second section related to diabetes, and the third section included questions related to COVID-19. The survey was written in English before being translated into Arabic (the mother tongue of all participants) and circulated through social media.

Sample size 

The sample size needed was calculated by Rasoft (Raosoft.com, 2022). Using a margin of error of ±4%, a confidence level of 95%, a 50% response distribution, and 71913.6 people, a sample size of 596 was required for obtaining data of statistical and biological significance.

Inclusion and exclusion criteria

All patients with type 1 and type 2 diabetes mellitus infected with COVID-19 were included in this study, including patients from both genders, all age groups and nationalities, and different smoking statuses in Saudi Arabia. This study excluded patients under 18 who did not confirm their infection with COVID-19 by polymerase chain reaction (PCR).

Statistical analysis

IBM Corp. Released 2013. IBM SPSS Statistics for Windows, Version 22.0. Armonk, NY: IBM Corp. used to perform the statistical analysis. Descriptive statistics were described as percentages for definite variables. The chi-square test was used to compare COVID symptoms with the type of diabetes. The analysis of the predictive factor for complications related to COVID-19 was performed by binary logistic regression analysis. A p-value considered statistically significant is less than 0.05.

## Results

We initially received a total of 916 responses to our online questionnaire. After determining the criteria (inclusion and exclusion), 781 patients were selected for the study. However, only 752 (96.3%) patients had DM, and 674 (86.3%) patients had contracted COVID-19. Thus, we included 674 participants in this study. Among the study population, 369 (54.7%) patients were female, 182 (27%) belonged to the 18-25 age group, and 230 (34.1%) were from the Western province of Saudi Arabia. Smoking was reported by 151 (22.4%) subjects, and 131 (19.4%) had multiple chronic diseases. T1D was comparatively more reported (51.3%) than T2D (Table 1).

**Table 1:** Basic characteristics of the participants N= number, %= percentage, DM= Diabetes mellitus

Participant’s characteristics		N	%
Have diabetes	No	29	3.7
	Yes	752	96.3
Infected with COVID-19	No	107	13.7
	Yes	674	86.3
Gender	Male	305	45.3
	Female	369	54.7
Age (years)	18-25	182	27.0
	26-35	134	19.9
	36-45	97	14.4
	46-55	132	19.6
	56-60	80	11.9
	>60	49	7.3
Province in Saudi Arabia	Central	161	23.9
	Eastern	85	12.6
	Northern	73	10.8
	Southern	125	18.5
	Western	230	34.1
Smoker	No	523	77.6
	Yes	151	22.4
Type of diabetes	Type 1 DM	346	51.3
	Type 2 DM	328	48.7
Chronic disease	Nothing	209	31.0
	Asthma	5	.7
	Cancer	2	.3
	Cardio-vascular disease	27	4.0
	Hypertension	151	22.4
	Hypothyroidism	15	2.2
	Renal disease	19	2.8
	Obesity	115	17.1
	Multiple diseases	131	19.4

Among the COVID-19-related symptoms in the participants, fatigue (65.6%) was the most frequently reported symptom, followed by headache (62.3%), cough (59.8%), loss of sense of taste or smell (49.4%), shortness of breath (43.6%), muscle pain (42.4%), and joint pain (39%). About 44% of patients reported high blood sugar levels, of which 25.5% had high fasting glucose levels (>126 mg/dL), and 18.5% had high postprandial glucose levels (>200 mg/dL). 4.2% of the participants had COVID-19 symptoms that lasted for more than three months. Similarly, 21.8% of patients were hospitalized due to these symptoms, and 9.6% were taken into the intensive care unit (Table 2).

**Table 2:** The prevalence of COVID-19-related symptoms in the patients with DM N= number, %= percentage, DM= Diabetes Mellitus

		N	%
Symptoms	Cough	403	59.8
	Anorexia	21	3.1
	Fever	5	0.7
	Loss of sense of taste or smell	333	49.4
	Chest pain	214	31.8
	Shortness of breath	294	43.6
	Fatigue	442	65.6
	Headache	420	62.3
	Digestion problems	143	21.2
	Distraction and memory problems	90	13.4
	Loss of appetite	250	37.1
	Psychological problems	99	14.7
	Joint pain	263	39.0
	Muscle pain	286	42.4
	Cramps	32	4.7
	Lymphadenitis	1	0.1
	Nothing	16	2.4
Blood sugar level before contracting COVID-19	Low: less than 70 mg/dL.	15	2.2
	Normal: Before eating (fasting): less than 100 mg/dL.	165	24.5
	Normal: After eating: less than 140 mg/dL.	197	29.2
	High: Before eating (fasting): Greater than 126 mg/dL.	172	25.5
	High: After meals: > 200 mg/dL.	125	18.5
Diabetic medications	Insulin	328	48.7
	Oral hypoglycaemic drugs	249	36.9
	Insulin and Oral hypoglycaemic drugs	97	14.4
Duration of symptoms after infection with COVID-19.	<1 month	414	61.4
	1 month	197	29.2
	3 months	35	5.2
	>3 months	28	4.2
Did your COVID-19 infection lead to hospitalization?	No	527	78.2
	Yes	147	21.8
Did your condition worsen, which led to your admission to intensive care?	No	621	92.1
	Yes	53	7.9

The distribution of COVID-19 symptoms according to the type of diabetes is provided in Table 3. The observation shows no statistically significant differences in the distribution of symptoms between the patients with two types of diabetes (p>0.05). When we compared the distribution of different types of complications with two types of diabetes, no significant differences were observed (p>0.05). However, we found that the persistence of COVID-19 symptoms for more than three months was comparatively more common in patients with T1DM (64.3%) than in T2DM (35.7%). However, this difference was not statistically significant. Additionally, the participants who were admitted to the ICU included patients with T1DM (58.5%) and T2DM (41.5%), p>0.05 (Table 4).

**Table 3:** The prevalence of COVID-19-related symptoms in patients with two types of DM N= number, %= percentage, * =chi square test

Symptoms		Type of diabetes		Total	P-value*
		Type 1	Type 2		
Cough	N	215	188	403	0.502
	%	53.3%	46.7%	100.0%	
Anorexia	N	15	6	21	
	%	71.4%	28.6%	100.0%	
Fever	N	2	3	5	
	%	40.0%	60.0%	100.0%	
Loss of sense of taste or smell	N	164	169	333	
	%	49.2%	50.8%	100.0%	
Chest pain	N	101	113	214	
	%	47.2%	52.8%	100.0%	
Shortness of breath	N	140	154	294	
	%	47.6%	52.4%	100.0%	
Fatigue	N	228	214	442	
	%	51.6%	48.4%	100.0%	
Headache	N	208	212	420	
	%	49.5%	50.5%	100.0%	
Digestion problems	N	71	72	143	
	%	49.7%	50.3%	100.0%	
Distraction and memory problems	N	51	39	90	
	%	56.7%	43.3%	100.0%	
Loss of appetite	N	127	123	250	
	%	50.8%	49.2%	100.0%	
Psychological problems	N	59	40	99	
	%	59.6%	40.4%	100.0%	
Joint pain	N	128	135	263	
	%	48.7%	51.3%	100.0%	
Muscle pain	N	139	147	286	
	%	48.6%	51.4%	100.0%	
Cramps	N	15	17	32	
	%	46.9%	53.1%	100.0%	
lymphadenitis	N	1	0	1	
	%	100.0%	0.0%	100.0%	

**Table 4:** The prevalence of COVID-19 complications in patients with two types of DM

			What type of diabetes do you have?		Total	P-value
			Type 1	Type 2		
Duration of symptoms after infection with COVID-19.	<1 month	N	222	192	414	0.119
		%	53.6%	46.4%	100.0%	
	1 month	N	89	108	197	
		%	45.2%	54.8%	100.0%	
	3 months	N	17	18	35	
		%	48.6%	51.4%	100.0%	
	>3 months	N	18	10	28	
		%	64.3%	35.7%	100.0%	
Hospitalized due to COVID-19.	No	N	276	251	527	0.308
		%	52.4%	47.6%	100.0%	
	Yes	N	70	77	147	
		%	47.6%	52.4%	100.0%	
Admitted to intensive care	No	N	315	306	621	0.278
		%	50.7%	49.3%	100.0%	
	Yes	N	31	22	53	
		%	58.5%	41.5%	100.0%	

To report the predictive factors for complications in diabetic people with COVID-19, logistic regression was performed. Participants who had persistent symptoms for more than three months and were hospitalized were considered to have complications. It was observed that age > 55 years [OR= 1.66 (1.01-2.56), p=0.045], the use of antidiabetic medications [OR= 2.10 (1.82-3.91), p=0.022], multiple comorbidities [OR= 3.21 (1.98-4.85), p=0.005], chest pain [OR= 2.54 (0.96-3.81), p=0.003], and joint pain [OR= 1.64 (0.73-2.94), p=0.025] were independently associated with COVID-19-related complications in diabetic patients (Table 5).

**Table 5:** Regression model for complications related to COVID-19 *= binary logistic regression

Predictive factors	Odds Ratio	95% Confidence Interval for OR		P-value*
		Lower Bound	Upper Bound	
Age > 55 years	1.66	1.01	2.56	0.045
Smoking	0.83	0.59	1.28	0.398
High blood sugar level before COVID	0.850	0.86	1.21	0.373
On insulin and Oral hypoglycaemic drugs	2.10	1.82	3.91	0.022
Multiple comorbidities	3.21	1.98	4.85	0.005
Cough	0.73	0.50	1.07	0.105
Anorexia	0.90	0.33	2.49	0.846
Fever	0.70	0.09	5.30	0.731
Loss of sense of taste or smell	1.04	0.72	1.50	0.831
Chest pain	2.54	0.96	3.81	0.003
Shortness of breath	0.72	0.49	1.05	0.088
Fatigue	1.36	0.89	2.07	0.152
Headache	1.55	1.04	2.30	0.030
Digestion problems	1.12	0.72	1.76	0.614
Distraction and memory problems	0.89	0.52	1.51	0.656
Loss of appetite	0.98	0.65	1.47	0.906
Psychological problems	0.68	0.41	1.13	0.137
Joint pain	1.64	0.73	2.94	0.025
Muscle pain	0.63	0.42	0.93	0.021
Cramps	0.81	0.35	1.84	0.607
Lymphadenitis	1.09	0.76	1.93	0.788

## Discussion

Evidence demonstrates that the COVID-19 pandemic also causes poor diabetes management, the progression of prediabetes to diabetes, an increase in the number of newly diagnosed diabetics, and a rise in corticosteroid-induced diabetes [20]. Diabetes that is poorly managed exacerbates the severity of COVID-19 and is linked to an increase in morbidity and mortality [23]. Microvascular and macrovascular problems, infection, and early death are all possible outcomes in diabetic patients, irrespective of the duration of the disease. Diabetic patients also exhibit impaired pulmonary function, peripheral airway obstruction, and lower carbon monoxide pulmonary diffusion capacity [24]. However, the implementation of effective treatments and management techniques requires a better understanding of the prevalence, type, and duration of persistent symptoms associated with COVID-19. The estimates of the prevalence and persistence of symptoms vary widely, possibly due to diverse study designs and disease classifications [25,26]. Recent research suggests that diabetic patients with COVID-19 who have comorbidities, older age, and obesity are more likely to have poor clinical outcomes and persistent symptoms following the resolution of the COVID-19 infection [27,28]. Our findings are in agreement with previous reports, indicating that the older age group (>55 years), the presence of multiple comorbidities, and the use of antidiabetic medications were associated with more persistent symptoms and complications. Experimental studies have yielded inconsistent results regarding the relationship between antidiabetic medications and COVID-19 severity and prognosis. The conflicting results in many clinical studies may partly be explained by ethnic and genetic differences, medical care accessibility, study setting and design, sample size, and the potential for adverse outcomes [29,30]. Increased COVID-19 infection severity has been associated with a dysfunctional endothelium, immune dysfunction, chronic low-grade inflammation, and a higher prevalence of pre-existing comorbidities [27].

Patients with DM may be more susceptible to acquiring COVID-19-induced adverse reactions because of shared pathology, such as heightened inflammatory response, blood sugar variability, and multi-organ injuries such as chronic kidney disease (CKD), neuropathy, brain damage, and cardiovascular diseases. Despite these commonalities, the underlying reasons for the increased susceptibility of DM patients to COVID-19 remain partly elusive. The COVID-19 infection induces an acute inflammatory response with hyperglycemia and tissue damage. In contrast, diabetes causes chronic, low-grade inflammation with poor glycaemic control and multi-tissue injury that progresses slowly over time, leading to the development of several complications [31,32]. Chronic inflammation in diabetic patients may be "locked and loaded" for virus-induced harm, fostering a negative feedback loop of cytokine production and hyperglycemic spikes that lead to multi-organ damage, particularly in organs already weakened by diabetic complications. Only a few studies have investigated the potential of pre-existing micro- and macrovascular problems in COVID-19 patients with high vulnerability to acute organ damage. It was reported that there was an independent relationship between pre-existing microvascular and macrovascular abnormalities and seven-day mortality, suggesting that people with DM are more likely to have poorer clinical outcomes [33]. In a multicenter study in China, it was reported that COVID-19 patients with DM were more likely to have pre-existing cardiovascular diseases, admission to ICUs, and develop acute complications such as acute cardiac injury, acute kidney injury, and acute respiratory distress syndrome, even though diabetes was not an independent risk factor for mortality due to COVID-19 [34]. Persistent symptoms and possible long-term consequences are becoming more apparent among COVID-19 survivors, drawing comparisons to the SARS and MERS-CoV outbreaks [34]. The long-term consequences of COVID-19 in diabetic patients are yet to be determined, but the evidence indicates that the pandemic has a significant load of persistent symptoms. COVID-19, like SARS-CoV and MERS-CoV, may aggravate and/or possibly cause cardiovascular dysfunction in T2DM patients [35,36]. Further, COVID-19 causes neurological symptoms and cognitive impairment, which share pathophysiology with diabetes via cytokine storm, hypercoagulability, and endothelial dysfunction [37]. Survivors of COVID-19 infection with diabetes may be at greater risk of developing long-term complications because of the pre-existing comorbidities that share pathophysiology with COVID-19-induced damage [37]. 

The current study has certain limitations. First, we used an online self-administered tool to record responses, which may have called upon social desirability and recall bias. Second, we overlooked some factors, such as DM severity or duration before COVID-19, which may lead to some confounding bias. Another limitation is that the study was conducted through a self-reported questionnaire, and DM diagnosis was based on participants input, which might lead to bias. Further research is warranted to determine the pathophysiology and prognosis of long-term persistent symptoms and complications in diabetic patients following the COVID-19 infection. 

## Conclusions

Persistent symptoms following the COVID-19 infection were common among patients with type 1 and type 2 Diabetes Mellitus. Fatigue, loss of sense or smell, and cough were the most common symptoms reported. Additionally, advanced age, use of antidiabetic medications, and the presence of multiple comorbidities were independently associated with COVID-19-related complications in diabetic patients.
